# Adult attachment styles and mothers’ life satisfaction in relation to eating behaviors in the families with overweight and obese children

**DOI:** 10.1371/journal.pone.0243448

**Published:** 2020-12-03

**Authors:** Agnieszka Pasztak-Opiłka, Romana de Jonge, Agnieszka Zachurzok, Małgorzata E. Górnik-Durose

**Affiliations:** 1 Faculty of Social Sciences, Institute of Psychology, University of Silesia, Katowice, Poland; 2 Department of Paediatrics and Paediatric Endocrinology, School of Medicine in Katowice, Medical University of Silesia, Katowice, Poland; Universidad de La Frontera, CHILE

## Abstract

Family plays a significant role in shaping children's eating behaviors. The aim of the study was to assess whether mothers’ attachment style, their life satisfaction and their own body weight can be associated with family eating behaviors. The results from 52 dyads (mothers/children) covered by the Metabolic Disease Clinic were analyzed. A targeted sample selection was used, taking into account the weight (overweight/obesity) and age (≥11 years) criteria of the child. The results have shown that the mother's body weight is a significant determinant of her child's body weight. The anxiety-ambivalent attachment style in mothers is a significant predictor of behaviors aimed at regulating and controlling affective states by food. A decrease in the knowledge of nutrition is associated with an increase in the level of anxiety-ambivalent and avoidant style. The avoidant attachment style is significantly associated with the nutrition organisation and control. Dysfunctional eating behaviors predominate among mothers with a lower level of life satisfaction. The lower the level of life satisfaction, the greater the tendency to regulate affective states and family relationships through nutrition, and to manifest improper organisation of nutrition. Mothers with obesity, compared to mothers with overweight and with normal body weight show a higher level of regulating emotions through food, improper organisation of nutrition and lower control in this area. The research results indicateshow significant relationships between insecure attachment styles, life satisfaction, and the mother's weight with eating behaviors unfavorable to health. It is therefore necessary to include family factors in the process of creating effective intervention strategies.

## Introduction

### Childhood overweight and eating behaviors

Childhood overweight and obesity has become a serious problem of public health [1,2]. From a global perspective, there is a worrying increase in the number of obese children and it is consistently indicated that children with overweight are predisposed to obesity in adulthood [3,4]. International research reports show that excess weight affects over 14% of girls and about 30% of boys aged 11–15 [5,6]. In the Silesian Province in Poland, the problem of overweight occurs in about 9% of children and adolescents aged 11–18, while obesity affects about 4% of representatives of this age group [7]. Psychological and environmental factors are increasingly exposed in analyses regarding the determinants of excessive body weight. Reports from the WHO research [4] indicate the important role of immediate environment in the development of childhood obesity and overweight.

Nutrition is one of the key factors affecting the development, functioning and health of an individual. Inappropriate nutrition, i.e. eating excessive amounts of food in relation to the actual needs of the body, and increased intake of calories in a portion of food, is one of the most important causes of obesity [8–12]. Proper eating habits include: eating regularly at least four meals a day at regular intervals, a diet rich in vegetables, fruits, and whole grain products, and limiting the consumption of highly processed products containing fats and sugars [13,14]. The following are considered by specialists to be improper eating habits: eating excess animal fats, sweets, salt, skipping meals, irregular eating habits, snacking between main meals and inadequate caloric value of consumed products [3,14].

Eating behaviors are formed from an early age. The parents’ role is to provide children with relevant knowledge and to model their behavior in relation to eating [15]. Numerous studies [16–18] indicate that the key role in nutrition is played by mothers, who are most often responsible for the diet quality, planning, organizing and preparing meals. Mothers play also a particularly important role in shaping and consolidating proper nutritional attitudes, beliefs, customs and the individual’s knowledge about the value of food [19–22]. Furthermore it is worth noting that the mother is usually one of the most important attachment figures for a child. The child shares a feeling of love with her, trusts her, she is an indisputable model and example, also for eating behaviors.

In this article we concentrate on certain determinants of eating habits, beliefs, attitudes and behaviors in families of a psychological nature. A number of factors are mentioned in the literature in relation to the family impact on childhood overweight and obesity [23]. Some studies show the association between attachment style, physical health and health-related behaviors [24–27], as well as between attachment style and various health disorders [25,28]. Also, many studies have confirmed that life satisfaction has a significant impact on eating behaviors and that parents of obese children present a lower level of life satisfaction [25]. Finally, as reported by Gibson et al. [29], the most important predictor of the child’s obesity is the mother's obesity.

### Attachment style

Ainsworth [30] defines attachment as a deep and enduring emotional bond that arises between two individuals across time and space. The parent-child attachment system formed in the dyad is a specific structure that develops a sense of trust in others [31,32]. Attachment developed in early childhood is seen as a matrix for later social relations [33]. According to the theory of Hazan and Shaver [34], adult attachment styles are defined in terms of a sense of security and trust in interpersonal relationships. The relationship between two people can be interpreted in the perspective of three attachment styles: secure, anxious-ambivalent and avoidant [32]. People with a secure attachment style create stable relationships, which are dominated by positive emotions, a high level of satisfaction and a sense of security in the relationship [28,35]. An anxious-ambivalent style is characterized by anxiety about the stability of the relationship, a reduced sense of security and oversensitivity to the relationship between self and partner. The avoidant style is manifested by the preference for clearly marked borders, the lack of tendency to establish open, close relationships with a partner and reluctance to closeness and confiding [32,35].

Generally, research results show the relationship between the attachment styles and physical health, preventive health behaviors such as physical activity and maintaining an appropriate diet [24,36] and the existence of links between attachment style and health disorders [25,28]. Research by McWilliams, Bailey [37] revealed also that secure attachment style is negatively associated with health problems such as depression and anxiety disorders. Moreover, studies on childhood obesity [26] indicate a significant role of the attachment styles of parents and their impact on the development and occurrence of obesity in children. Parents of children with normal body weight are more likely to present a secure attachment style in comparison to parents of overweight and obese children [25].

Results show that the incidents of irregularities in the control of nutrition, diet quality, planning and organisation of eating behaviors occur mainly in people with insecure attachments [13,26,38]. A number of previous studies [e.g. 26,27,38,39] suggest that people with insecure attachment apply negative regulatory strategies in stressful situations and regulate their affect through eating. The emotional states that arise from parent-child interactions can influence eating behaviors. Secure attachment of parents is a source of availability and emotional support for children. A secure attachment enables the child to express and experience his or her emotions. Parents in secure relationships are consistent in their behavior and sensitive to the stimuli coming from their children, they have a repertoire of adaptive and flexible strategies for dealing with new and stressful situations. Therefore, there is no need to use food to regulate the affective states of both parents and children.

### Life satisfaction

The sense of life satisfaction is associated with the concept of happiness, which is one of the most important determinants of human behavior. According to Shin and Johnson [40], the evaluation of life satisfaction is made by a global assessment of an individual’s life according to their valued criteria. The appraisal of life satisfaction results from a comparison of one's own situation with individually set standards [41]. It is a valuable resource influencing psychosocial health [42]. Life satisfaction is associated with health behaviors, including eating behaviors [42–44]. A higher level of life satisfaction goes along with healthy eating habits [45–47]. Results of a study by Kuroki [46] indicate the negative correlation between overweight and obesity and life satisfaction. Overweight and obese people were less satisfied with their life than respondents with adequate body weight. The study by Strine et al. [47] proved that as the level of life satisfaction decreases, the risk of unhealthy behaviors, smoking, obesity increases and the frequency of physical activity decreases. The risk of obesity among people with low levels of life satisfaction was 2–3 times higher compared to people with a high level of life satisfaction.

Furthermore, positive social relationships are a significant source of life satisfaction. The study by Kaleta i Mróz [48] shows that a sense of support and attachment to other people is conducive to taking positive health behaviors. Social support received from loved ones on a general level positively correlates with positive mental attitudes and proper eating habits. In turn a lack of satisfaction in interpersonal relationships, a lack or low level of social support are associated with reduced physical activity, increased smoking frequency and inadequate nutrition. In the above context, the high quality of partnership–as one of the determinants of overall life satisfaction–appears to be directly linked to health. Generally, research reports [44,49] show that positive partnerships reduce the probability of risky eating habits. Consequently, the higher the level of quality of marital functioning and the associated level of life satisfaction, the higher the level of health and the lower the risk of health problems such as depression, cardiac problems or diabetes.

### The current study

The purpose of the present study is to investigate the relationship between eating behaviors and certain characteristics of mothers of children with excessive body weight. In our research, we focus on mothers, as they play a key role in family nutrition. As the main caregivers they are most often responsible for planning, organising and preparing meals. Furthermore, mothers spend a lot of time with their children, model their various behaviors and, consequently, strongly influence their eating habits [29]. Previous studies [18,50] show also that as the mother's weight increases, her child's weight increases too. We find particularly interesting the relationship between mothers’ weight, mothers’ attachment style, their life satisfaction and their eating behaviors, because these issues have not been examined together in the empirical research thus far. Defining especially the relationship between mothers’ attachment style, life satisfaction and eating behaviors can make an important contribution to implementing preventive measures, setting trends in the modification of nutrition-related behaviors and monitoring changes in health promoting eating practices.

## Materials and methods

### Participants and procedures

The research was conducted under the Agreement on Scientific and Research Cooperation, of 6 July 2016, No. KNM/819/2016, between the Medical University of Silesia and the University of Silesia, in cooperation with the Upper Silesian Children's Health Centre in Katowice. The study procedure was approved by the Bioethics Committee of the Medical University of Silesia (Approval No. KNW/0022/KB/70/16) and the Ethics Committee of the Scientific Research of the Faculty of Pedagogy and Psychology of the University of Silesia in Katowice (Approval No. 3/2018).

Sixty five dyads—mothers and their children, covered by the specialist care of the Metabolic Disease Clinic of the Upper Silesian Children's Health Centre in Katowice, joined the study. A purposive sample was selected, taking into account the weight (overweight/obesity) and age (≥11 years) criteria of the child. Participation was fully voluntary and anonymous, respondents were informed about the possibility to withdraw from the research at any time. Mothers and children agreed to participate in the study by signing a written consent. Mothers independently filled out questionnaires in paper or electronic versions (according to their preferences), comfortable conditions and separate rooms were provided. Mothers declared their weight and height. The children were weighed and measured by an endocrinologist, and then qualified for the examination on the basis of a medical diagnosis based on centile grids (overweight: body weight of 90–97 percentile; obesity with a body weight above 97 percentile). Some data were rejected at the beginning due to significant gaps in relevant information (omitted, incorrectly answered questionnaire items).

The final statistical analysis was performed on 52 mothers aged 32–56 years (*M* = 41.81, *SD* = 5.21) of children aged 11–18 years (*M* = 14.08, *SD* = 2.12). Although the studied group was small, it allowed identifying strong effect sizes with statistical power of .80. All children were characterized by excessive body weight. Based on percentile grids, 19% of children were overweight (>90 percentile) and 81% were obese (>97 centile). In the study group overweight (27%) and obese (42%) mothers predominated, only 31% of mothers had normal body weight (BMI min. = 20.45, max. = 42.52, *M* = 28.59, *SD* = 5.36).

## Methods

The conducted study was cross-sectional. Data were obtained from mothers of children with excessive body weight by self-report methods. The following questionnaires were used in the study:

1. Questionnaire of Attachment Styles (KSP), by M. Plopa [51]. It is a tool for diagnosing the attachment style in a romantic relationship (i.e. partnerships, marriages). It consists of 24 statements, arranged in three scales, related to three attachment styles: secure (α = 0.91), anxious-ambivalent (α = .78) and avoidant (α = .80), which are described in the introductory section of this paper. The surveyed person is asked to respond to each statement on a seven-point scale (1- *strongly disagree*, 7- *strongly agree*).
2. Satisfaction with life scale (SWLS), by E. Diener, R.A. Emmons, R.J. Larson, & S. Griffin, in the Polish adaptation by Juczynski [52]. SWLS is used to measure the overall level of satisfaction with life (α = .81). It contains five statements, respondents make a subjective assessment on the scale (1- *completely disagree*, 7- c*ompletely agre*e), to what extent each of them relates to their current life.
3. Eating behaviors questionnaire (KZZ) is the pilot version (in validation) created by the first author of this paper to evaluate family eating behaviors. The questionnaire measures eating behaviors and certain factors related directly to eating habits (e.g. various attitudes and beliefs about healthy diet as well as nutrition-related practices). The initial items of the Eating behaviors questionnaire were selected mainly from descriptions of family eating habits and behaviors obtained from parents of children treated for obesity in the Metabolic Disease Clinic of the Upper Silesian Children's Health Centre in Katowice. The final version final version of KZZ questionnaire consists of 39 items to which the respondent answers on a Likert type scale (1-*definitely not*, 5-d*efinitely yes*). A principal components analysis was carried out on the data obtained with the questionnaire. The Varimax rotation was applied. The Kaiser-Meyer-Olkin measure–KMO = .823 showed that the data were well suited for such an analysis. Also the Bartlett’s test of sphericity was significant (chi square = 10862.43, df = 4278, p < .001), which supported the presence of correlations between the items.
  Six factors were identified, and they explained 42% of total variance. The factors formed the basis for six subscales relating to two positive (knowledge of nutrition, control of nutrition) and four negative (negative beliefs and cultural customs, regulation of family relationships, regulation of emotions, incorrect organisation of nutrition) eating behaviors. The scale of *negative beliefs and cultural customs* (α = .70; 7 items) is based on stereotypes and misconceptions about proper nutrition and sources of obesity, diminishing one’s own role in its formation and attributing responsibility to environmental and genetic factors (e.g. "Genes are primarily responsible for obesity," or "I think advertised products are healthier than those unknown."). The second scale–*knowledge of nutrition* (α = .77; 6 items) is based on the knowledge of the principles of healthy eating, quality and value of consumed products (e.g. "We read labels carefully, becoming familiar with the value of the consumed meals", "We try to make meals healthy and low in calories."). The third scale relates to a *regulation of family relationships through nutrition* (α = .71; 8 items). It refers to the relationships in the family, views on the implementation of dietary restrictions, the consequences of compliance with them, control of nutrition, also raises the problem of motivation to apply dietary recommendations and their impact on the emotional state of the family (e.g. "The introduction of a diet in our home creates a lot of tension and quarrels," "It happens in our family that one of the parents or other family members forbid the children sweets or unhealthy meals, and the other secretly gives them to them", "Taking sweets away the child from is one of the basic punishments used in our family"). The next scale concerns the area of *regulation of emotion through eating* (α = .73; 7 items). It refers to regulation of emotions and stress by means of food, e.g. "Food affects my well-being", "In stressful situations, food allows me to calm down"). The fifth scale is the *improper organisation of nutrition* (α = .75; 6 items). The items that make up this scale illustrate how the respondent's nutrition is organized, the amount of time spent on eating, the regularity, quality, quantity and manner of meals consumed (e.g. "Due to lack of time, we give up a meal during the day," "Every household member can eat when they are hungry, regardless of the meal times"). The *control of nutrition* scale (α = .42; 5 items) includes items that help see whether weight control is practiced in the family, and whether caregivers have an impact on the quantity and quality of meals consumed by a child (e.g. "We make sure that our child does not eat too much", "In our family we constantly monitor the body weight, weighing regularly" or "I think that the child knows best what is good for him/her").
4. The one-item scale was used to assess the family financial situation. The respondents assess their own material standard of living on a 5-point scale from 1 –*very poor* (not enough to satisfy basic needs) to 5 –*very good* (able to afford a comfortable life).
5. Survey—containing questions about age, sex, height and weight, education, family financial situation and the respondent’s family structure.

The test results obtained were statistically developed using the SPSS Statistic 25 program. Pearson's r-correlation analysis, ANOVA analysis of variance, post hoc: Tuckey test and Games-Howell test, Mann-Whitney U test, Student's t-test and multiple regression analysis (the input method) were used.

## Results

Characteristics of the sample and descriptive statistics of the key variables addressed in the study are presented in Table 1.

**Table 1:** Characteristics of the sample and descriptive statistics of the key variables.

Variable		*N (%)*
**Mother’s education**	Higher	13 (25)
	Secondary	22 (42.3)
	Vocational	17 (32.7)
**Financial situation**^**a**^	Very good financial situation	4 (7.7)
	Good financial situation	20 (38.5)
	Average financial situation	22 (42.3)
	Poor financial situation	6 (11.5)
**Family structure**^b^	Full family	39 (75)
	Incomplete family	13 (25)
	Small family	42 (80.8)
	Large family	10 (19.2)
		***Mean* (*SD*)**^c^
**Attachment style**	Secure	42.23 (10.65)
	Anxious- ambivalent	25.92 (12.91)
	Avoidant	20.37 (9.44)
**Life satisfaction**		21.42 (4.67)
**Eating bahaviours**	Negative beliefs and cultural customs	16.38 (3.44)
	Knowledge of nutrition	13.63 (3.06)
	Regulation of family relationships	15.21 (4.32)
	Regulation of emotions	17.17 (3.91)
	Improper organisation of nutrition	12.12 (2.82)
	Control of nutrition	10.50 (2.20)

^a^in The Scale of Assessment of the Family Financial Situation.

^b^Full family: Mother + father/stepfather + child/children; incomplete family: Single mother + child/children; small family: 1 or 2 children; large family: 3 or more children.

^c^M-mean, SD- standard deviation.

### Mothers' weight and eating behaviors

The preliminary analysis revealed a positive correlation between mothers and their children's body weight (r = .28, p = .046).

In order to assess differences in mothers' eating behaviors depending on the mothers’ weight, ANOVA (and ANOVA Welch with correction for variance heterogeneity) was performed together with post-hoc tests (Tuckey or Games-Howell depending on the ANOVA variant). Due to the introduction of the grouping factor, the above analyses were preceded by the evaluation of the distribution of variables. In the group of mothers with normal body weight subscale "knowledge of nutrition" and "regulation of relationships in the family" did not have a normal distribution. However, it is worth noting that in the case of these variables, the skewness and kurtosis did not exceed the conventional absolute value of 2, so the decision to use parametric analysis (in this case ANOVA) was maintained. The results are presented in Table 2.

**Table 2:** Differences between eating behaviors in families depending on the mothers’ weight.

Variable	Negative beliefs and cultural customs M (SD)/test		Knowledge of nutrition M (SD)/test		Regulation of relationships in the family M (SD)/test		Regulation of emotions M (SD)/test		Improper organisation of nutrition M (SD)/test		Control of nutrition M (SD)/test	
**Mothers’ weight**^a^												
1-Normal weight (n = 16)	15.38 (2.90)	F = 2.15^b^ p = .128	14.88 (3.44)	F = 3.38 p = .042*^c^ ω^2^ = .08^d^ post hoc: 1>3*	14.50 (4.29)	F = .52 p = .595	15.19 (2.66)	F = 3.28 p = .046* ω^2^ = .12 post hoc: 1<3**	11.38 (2.45)	F = 6.14 p = .004* ω^2^ = .17 post hoc:1<3* 2<3**	11.25 (1.84)	F = 6.81 p = .002* ω^2^ = .18 post hoc: 1>3** 2>3**
2-Overweight (n = 14)	15.79 (2.94)		14.07 (2.89)		14.93 (4.84)		17.86 (4.74)		10.71 (2.55)		11.50 (1.87)	
3-Obese (n = 22)	17.50 (3.89)		12.45 (2.52)		15.91 (4.08)		18.18 (3.71)		13.55 (2.67)		9.32 (2.15)	

^a^Normal weight BMI = 18.5–24.9, overweight BMI = 25–29.9, obesity BMI> 30.

^b^F- ANOVA test statistics.

^c^p- value

*p<0.05

**p<0.01.

^d^ω2—omega-square (measure of effect strength), post hoc: Tuckey test or Games-Howell test.

The results that are presented in Table 2 show that mothers' body weight plays an important role in eating behaviors. In families with obese mothers, in comparison to overweight mothers and mothers with normal body weight, the organisation of nutrition was significantly worse. Compared to mothers with normal body weight, obese mothers reported regulating emotions through eating more often. Generally women with normal body weight, compared to obese women, had a better knowledge of nutrition and a higher level of the control of nutrition- which can be perceived as important determinants of proper eating behaviors. In the area of nutritional control, overweight mothers also did better than obese mothers.

### Satisfaction with life in mothers and eating behaviors in their families

In order to answer the question about the relationship between life satisfaction and eating behaviors the Pearsons’ r- correlation analysis was performed. The analysis (see Table 3) revealed that the lower the level of life satisfaction, the greater the tendency to regulate affective states and family relationships through nutrition and to manifest the negative behaviors regarding organisation of nutrition. In addition, an increase in the satisfaction with life promoted an increase in the knowledge of nutrition and the control of nutrition.

**Table 3:** Correlations of parents’ attachment styles, life satisfaction and family eating behaviors^a^.

Variable	Negative beliefs and cultural customs	Knowledge of nutrition	Regulation of family relationships	Regulation of emotions	Improper organisation of nutrition	Control of nutrition
**Attachment style**						
Secure style	-.16	.16	-.26	.09	-.06	.04
Anxious- ambivalent style	.19	-.32*	.49**	.44**	.25	-.20
Avoidant style	.36**	-.31*	.40**	.19	.32*	-.30*
**Life satisfaction**	.05	.39**	-.46**	-.35*	-.42**	.35*

^a^R- Pearson correlation analysis

*p < .05

**p < .01.

### Maternal attachment styles and eating behaviors in the family

To answer the question about the relationship between maternal attachment style and eating behaviors in the family, the Pearson’s r- correlation analysis was conducted. It showed significant associations between attachment styles of mothers and eating behaviors in their families. The lower the level of anxiety-ambivalent and avoidant attachment styles, the greater the nutritional knowledge. There was a positive association between insecure attachment styles and the tendency to regulate family relationships through food. In addition, an increase in the level of anxiety -ambivalent style promoted an increase in the tendency to regulate and control the emotions through acts of consumption. The higher intensity of the avoidant attachment style, the greater the tendency in negative beliefs and cultural customs, and improper organisation of nutrition and control of nutrition (Table 3).

### Predictors of eating behaviors in families of children with excessive body weight

In order to indicate significant predictors of eating behaviors in families of children with excessive body weight, a regression analysis (the input method) was performed for each of the eating behaviors. Mother’s BMI, three attachment styles, and mother’s satisfaction with life were introduced into models as potential predictors. Each model was verified in terms of the assumptions required to apply regression analysis (normality of residue distribution, autocorrelation of residuals, homoscedasticity, collinearity).

The results are presented in Table 4.

**Table 4:** Regression analysis of predictors of eating behaviors^a^.

Variable	Negative beliefs and cultural customs			Knowledge of nutrition			Regulation of relationships in the family			Regulation of emotion			Improper organisation of nutrition			Control of nutrition		
	F = 2.35*			F = 3.55**			F = 5.44**			F = 4.10**			F = 4.00**			F = 3.75**		
	R^2^ = .12			R^2^ = .20			R^2^ = .30			R^2^ = .23			R^2^ = .23			R^2^ = .21		
	**ß**	**t**	**p**	**ß**	**t**	**p**	**ß**	**t**	**p**	**ß**	**t**	**p**	**ß**	**t**	**p**	**ß**	**t**	**p**
**Maternal BMI**	.23	1.72	.092	-.27	-2.10	.041	.04	0.30	.766	.24	1.88	.066	.27	2.14	.038	-.33	-2.56	.014
**Secure style**	-.03	-0.24	.810	.08	0.56	.580	-.18	-1.28	.208	.12	0.88	.383	.07	0.53	.596	-.09	-0.66	.509
**Anxious- ambivalent** style	.01	0.07	.942	-.10	-0.58	.564	.31	2.01	.050	.37	2.31	.025	-.06	-0.39	.696	.12	0.77	.448
**Avoidant style**	.34	1.88	.066	-.11	-0.64	.524	.08	0.48	.632	-.06	-0.38	.704	.25	1.50	.139	-.28	-1.65	.106
**Life satisfaction**	.17	1.21	.231	.28	2.06	.045	-.30	-2.39	.021	-.20	-1.53	.132	-.35	-2.64	.011	.30	2.21	.032

^a^F- statistics for F model; R^2^ coefficient of determination; ß- coefficient of regression; t- test statistics; p-value.

The predictors explained between 12% (negative beliefs and cultural customs) and 30% (regulation of relationships in the family) of variance of various aspects of eating behaviors. In the case of the first factor–negative beliefs and cultural customs–the strongest positive predictor was the avoidant attachment style (β = .34), followed by mothers’ BMI (β = .23; both not significant). In the case of the knowledge of nutrition the strongest positive predictor was mothers’ life satisfaction (β = .28) and negative–their BMI (β = -.27). In the case of regulation of relationships in the family, there were two significant predictors–the anxious-ambivalent attachment style (β = .31) and life satisfaction (β = -.30). The regulation of emotion was significantly predicted by the anxious-ambivalent attachment style (β = .37). The improper organisation of nutrition was explained mainly by mothers’ life satisfaction (β = -.35) and their BMI (β = .27). Mothers’ BMI (β = -.33) and their life satisfaction (β = .30) were significant predictors of the control of nutrition.

Of all predictors life satisfaction seemed to be the most prominent–it was significant in most cases. The higher the life satisfaction in mothers, the better the knowledge and control of nutrition in the family. Lower life satisfaction on the other hand went along with stronger tendencies to regulate relationships through eating and improper organisation of nutrition in the family. High maternal BMI explained significantly the improper organisation of nutrition. Among attachments style the most important was the anxious-ambivalent which significantly predicted the regulation of relationships and emotion through eating. The avoidant style went along with the negative beliefs and cultural customs.

## Discussion

The main purpose of the study was to investigate the relationship between eating behaviors in families with children with excessive body weight and certain characteristics of mothers–their attachment style in partner relationships, their life satisfaction and BMI.

The research results have been consistent with previous reports that emphasize the relationship between the attachment style presented by parents and nutrition-related practices [26,27,38,53]. The attachment style forms a base for human cognitive activities, affects the formation of emotions, and the ways of controlling them, the quality of interpersonal relationships and health [39]. It is therefore inherently associated with expectations, desires, emotions and social behaviors [53] The results obtained show that insecure styles may have a negative impact on the activities and behaviors related to proper nutrition. The insecure attachment is associated with tendency to regulate emotions and relationships through consumption acts. The results obtained are in line with previous reports [26,27,38,39] indicating that people with insecure attachment can use negative regulatory strategies in stressful situations, internalize encountered difficulties and regulate the affect through food and isolate themselves from the environment.

People with insecure attachment experience more problems with emotions and adaptation to new situations [39]. The results of the current study reveal that the anxiety-ambivalent attachment is an important predictor of emotional eating–the more intense its level, the greater the person's tendency to eat extensively in order to reduce stress or to improve one’s emotional state. The avoidant attachment style on the other hand is associated with poor organisation and control of nutrition. This result corresponds with previous studies [13,26,38], indicating the occurrence of irregularities in the control of nutrition, diet quality, planning and organisation of eating behaviors in people with the insecure attachments.

The research considered also the relationships between life satisfaction and eating behaviors. Previous analyses [54,55] have shown that the low level of life satisfaction encourages regulating family relationships and emotional states through food. The obtained results correspond to these reports confirming that dysfunctional eating behaviors predominate in people with lower life satisfaction. Satisfied people show a positive attitude to life, greater motivation to act, and in adverse situations usually take active strategies to solve encountered problems. In contrast, people declaring low life satisfaction have a higher rate of negative emotions, lower level of quality of life, motivation and optimism, and in stressful situations more often apply passive or avoidant coping strategies. People with the low level of life satisfaction may tend to regulate well-being and interaction in the family environment through nutrition [45–47].

In the present study the correlation analyses also showed that the high level of life satisfaction is advantageous to the higher level of knowledge and control of nutrition, which is in accordance with previous reports [45–47]. It can therefore be assumed that people satisfied with their current life will have a greater motivation to take action for health, planning and organizing nutritional behaviors, as well as broadening knowledge about proper nutrition.

It is worth noting that the regression analysis showed that in relation to the knowledge of nutrition, life satisfaction turned out to be the most important predictor. Referring to the results obtained, it can be concluded that the high level of life satisfaction will be beneficial to proper eating habits, based on the knowledge of the principles of healthy eating and care about the nutrition value of consumed food.

One of the results of the current study, which is also in line with the previous [18,50], show positive correlation between mother's and her child's weights. Furthermore, it has been observed that mothers with normal body weight are characterized by a higher level of positive eating behaviors compared to mothers with excessive body weight. It turned out that obese mothers, compared to mothers with normal body weight, more often control and regulate their emotions through consumption acts. This group is also characterized by a higher level of improper organisation of nutrition and lower control in this area. The results obtained coincide with previous studies [56,57] indicating that people who have weight problems in the face of challenges, difficult situations and stressors more often use negative coping strategies and regulate emotional states using food. Maternal BMI is a significant predictor of negative eating behaviors undertaken to regulate affective states. The BMI also explained the level of the knowledge of nutrition. It is in line with previous reports that indicate that obese mothers have a lower level of knowledge about healthy eating compared to mothers with normal body weight [58,59].

## Conclusions

Research results revealed significant relationships between insecure attachment styles and improper eating behaviors. A decrease in the knowledge of nutrition was associated with an increase in the level of anxiety-ambivalent and avoidant attachment styles. In addition, the anxiety-ambivalent style turned out to be a positive, significant predictor of eating behaviors aimed at regulating and controlling affective states. It means that in order to improve the impact and intervention in therapeutic work with a family with obesity, it is necessary to take into account the adult attachment styles of parents.

A high level of life satisfaction manifests itself as a motivating factor for acquiring and expanding the knowledge about nutrition and it is conducive to the proper organisation and control of nutrition. Life satisfaction can be a valuable personal resource in the scope of shaping and strengthening motivation to change in the area of the treatment of obesity.

Women with obesity present greater disorganisation of nutrition practices; they also more often regulate their emotions with food. Women with normal body weight have better nutrition knowledge and control. It indicates deficits in self-control as well as in effective and constructive strategies of affect regulation, especially in the group of obese women.

The mother's body weight correlates with her child's body weight. In addition, it is also one of the most important factors connected with the organisation of nutrition. It is therefore crucial for the potential interventions (e.g. therapeutic, dietary) to have a systemic character and cover the entire patient's family system.

## Limitations and future directions

The present study has some limitations. First, the clinical group of mothers was small. However it allowed identifying strong effect sizes with statistical power of .80. Secondly, the study group was homogeneous in terms of gender with a socioeconomic middle class predominance. In further exploration, to obtain a broader perspective of relationships and conditions, it would be advisable to include men as fathers or guardians of obese children. Another limitation of the research is the fact that the respondents themselves declared their weight and height. From the perspective of psychosocial functioning, one should take into account potential distortions, discrepancies and irregularities of the data, caused by the operation of social mechanisms of self-presentation, which may involve the provision of incorrect or untrue information about oneself. In subsequent studies, independent measurements of these variables should be carried out to increase the level of reliability of the results obtained. Another important limitation is the fact that the study was conducted in the Polish culture, so caution should be exercised in generalizing results on an international scale.

The limitation in relation to the tools used is also noteworthy. The Eating Behaviors questionnaire used in the research was a pilot version, still in the validation process. In the perspective of future research, it would be advisable to use optional other methods (e.g. structured interview). As part of broadening the perspective of the studied relationships between variables, in further analyses, it is also worth considering a broader picture of factors that may be associated with the attachment style and life satisfaction (e.g. self-esteem, coherence, subjective sense of control, sense of self-efficacy; and in aspect functioning of the family system—level of communication or consistency).

Despite the aforementioned limitations, the conducted research has significant value in the application and theoretical areas. The results bring a new look at the family system from the perspective of parents' relationships and life satisfaction, and relate these dimensions to eating behaviors. By referring to the theory of attachment and life satisfaction, research shows how important these variables are for the dimension of behavior related to health and nutrition. Based on the results obtained, in future work with the patient, it is worth considering the factor of life satisfaction with its connection with motivation, which optimal level is key in the process of change. It should also be remembered that satisfaction with life is associated with a sense of self-efficacy, which, being an important resource of the individual, is related to the undertaking and implementation of set goals, perseverance in achieving them and the ability to take appropriate strategies to cope with changes [2,60]. Consequently, the aspect of family functioning and parents’ relationship should be taken into consideration when creating and designing effective strategies, intervention and preventive programs.

## Supporting information

S1 File (DOCX)
