# Supplementary material for: Adult attachment styles and mothers’ life satisfaction in relation to eating behaviors in the families with overweight and obese children
Source: PLoS One. 2020 Dec 3;15(12):e0243448. doi: 10.1371/journal.pone.0243448 (PMC7714109; doi:10.1371/journal.pone.0243448)
Supplement: S1 File — (DOCX) [file pone.0243448.s001.docx]

**Eating Behaviours Questionnaire**

Please read the following statements and tick the boxes most appropriate to your eating behaviours and attitudes.

**1**- **definitely no**

**2**- **rather no**

**3**- **difficult to say**

**4**- **rather yes**

**5**- **definitely yes**

1. Introducing a diet for all family members is not fair to those, that don’t require it 1 2 3 4 5

2. We consume mainly natural or organic products 1 2 3 4 5

3. We usually eat meals together at the table 1 2 3 4 5

4. Food affects my mood positively 1 2 3 4 5

5. In my opinion a child knows best what is good for him 1 2 3 4 5

6. Due to lack of time we give up some meals during the day 1 2 3 4 5

7. Prohibiting or restricting food for a child is a form of child abuse 1 2 3 4 5

8. We always have sweets at home in case of any unannounced guest visit 1 2 3 4 5

9. We study the nutrition labels carefully 1 2 3 4 5

10. We hide sweets from children or other family members 1 2 3 4 5

11. Giving up sweets is difficult for our family 1 2 3 4 5

12. In our family we are constantly monitoring our body weight 1 2 3 4 5

13. Consuming food in stressful situations calms me down 1 2 3 4 5

14. Parents should reprimand the child when he eats too little 1 2 3 4 5

15. We try to do our best to keep the meals healthy and low in calories 1 2 3 4 5

16. The introduction of a diet in our family raises many tensions and quarrels 1 2 3 4 5

17. It is common for hungry people to feel sad 1 2 3 4 5

18. In our family we all consume the same meals 1 2 3 4 5

19. We eat 5 meals a day 1 2 3 4 5

20. Genes are primarily responsible for obesity 1 2 3 4 5

21. Snacking in between meals is common in our family 1 2 3 4 5

22. It happens, in our family, that one of the parents or other family members forbids the

child from sweets or unhealthy meals, and the other secretly gives them to him 1 2 3 4 5

23. We enjoy coffee and sweets following a dinner 1 2 3 4 5

24. Our family eats meals in front of the TV or computer 1 2 3 4 5

25. We pay attention to our child that he doesn't eat too much 1 2 3 4 5

26. In our home sweets are easily accessible 1 2 3 4 5

27. We pay a lot of attention to healthy food 1 2 3 4 5

28. Our daily meals are at fixed times 1 2 3 4 5

29. If I promise my child a snack, he will eat his meal better 1 2 3 4 5

30. It is pleasant to snack while reading a book 1 2 3 4 5

31. Beside the regular meals, each family member can eat whenever he/she is hungry 1 2 3 4 5

32. Being healthy is associated with being slim 1 2 3 4 5

33. We try to ensure that our child is never hungry 1 2 3 4 5

34. We are aware of calorie value of each meal consumed at home 1 2 3 4 5

35. It happens, that I lose control over what we eat at home 1 2 3 4 5

36. Eating more than planned makes me feel guilty 1 2 3 4 5

37. In my opinion the advertised products are more healthy than unknown 1 2 3 4 5

38. Taking the sweets away from child, is one of the basic punishment methods that we apply 1 2 3 4 5

39. In our family we often skip breakfast 1 2 3 4 5

**Scoring of the Eating Behaviours Questionnaire**

1. **Negative beliefs and cultural customs: 1, 8, 14, 20, 26, 33, 37**
2. **Knowledge of nutrition: 2, 9, 15, 21*, 27, 34**
3. **Regulation of family relationships through nutrition: 3*, 10, 16, 22, 29, 35, 38, 18**
4. **Regulation of emotion through eating: 4, 11, 17, 23, 30, 36, 13**
5. **Improper organization of nutrition: 6, 19*, 24, 31, 39, 28**
6. **Control of nutrition: 7*, 12, 25, 5, 32**

*****Reversed items
